# Organic manure input improves soil water and nutrients use for sustainable maize (*Zea mays*. L) productivity on the Loess Plateau

**DOI:** 10.1371/journal.pone.0238042

**Published:** 2020-08-25

**Authors:** Xiaolin Wang, Jiakun Yan, Xiong Zhang, Suiqi Zhang, Yinglong Chen

**Affiliations:** 1 College of Life Sciences, Yulin University, Yulin, Shaanxi, PR China; 2 State Key Laboratory of Soil Erosion and Dry Land Farming on the Loess Plateau, Northwest A&F University, Yangling, Shaanxi, PR China; 3 The UWA Institute of Agriculture, and School of Agriculture and Environment, The University of Western Australia, Perth, WA, Australia; Universidade de Santiago de Compostela, SPAIN

## Abstract

Long-term chemical fertilizer input causes soil organic matter losses, structural compaction, and changes in soil water and nutrient availability, which have been subdued in the most of dry farmland in China. The concept of “more efficiency with less fertilizer input” has been proposed and is urgently needed in current agriculture. Application of chemical fertilizer combined with organic manure (OM) could be a solution for soil protection and sustainable production of dry-land maize (*Zea mays*. L). Field research over three consecutive years on the Loess Plateau of China was conducted to evaluate the integrated effects of chemical fertilizer strategies and additional OM input on soil nutrients availability and water use in maize. The results showed that, after harvest, soil bulk density decreased significantly with OM application, concomitant with 11.9, 18.7 and 97.8% increases in topsoil total nitrogen, organic matter, and available phosphorus contents, respectively, compared with those under equal chemical *NPK* input. Water use in the 1.0–1.5 m soil profile was improved, therefore, the soil conditions were better for maize root growth, leaf area and shoot biomass of individual maize plants increased significantly with OM application. Optimized *NPK* strategies increased grain yield and water use efficiency by 18.5 and 20.6%, respectively, compared to only chemical *NP* input. Furthermore, additional OM input promoted yield and water use efficiency by 8.9 and 5.8%, respectively. Addition of OM promotes sustainable soil and maize grain productivity as well as friendly soil environmental management of dry land farming.

## Introduction

In the last 30 years, grain production in China has increased by 71%, which was supported by a 271% increase in chemical fertilizer input [1]. Excessive inputs of chemical nitrogen (*N*) and phosphorous (*P*) inevitably cause soil degradation and environmental imbalances [2–5]. Furthermore, soil natural nutrient reservation has weakened and impeded sustainable soil productivity [6–8], and low nutrient and water use efficiency followed [9, 10]. Currently, grain production is moving towards the efficient use of resources and sustainable yield increases with low environmental costs. Thus, the efficient and sustainable management of fertilizer use must be explored [10, 11].

Producing more grain with less fertilizer is a daunting but necessary task in China [12]. Decades of field tests have produced two approaches that can almost double maize yield without the use of more N fertilizer [13, 14]. Specifically, these methods are the integrated soil-crop system management system (ISSM) and in-season root-zone N management strategy (IRNM), which are designed according to the local environment, appropriate crop varieties, sowing dates, planting densities and advanced nutrient management to help enhance the understanding of eco-physiological properties and soil biogeochemistry [3, 14]. Pinitpaitoon et al. [15] report that cultivation practices with organic manure improve soil properties, balance soil organic matter (*SOM*), *N* and *P* retention and availability [7, 11], and finally, increase soil water use efficiency in dry-land farming [16, 17]. Tillage systems with organic and inorganic N sources enhance the mineralization/decomposition rates of organic residues, which are essential for crop growth [16–18]. In recent maize cultivation, organic manure has been widely used for its agronomical and environmental benefits [19], and it brings the maize yield close to its biological potential while reducing the input of chemical fertilizer [20]. Therefore, soil nutrients storage and crops uptake are enhanced by the additional organic manure input, subsequently ensuring a higher grain yield with less chemical fertilizer and environmental costs [18, 21]. However, when additional organic manure is applied, how soil water and nutrients are transferred remains unclear in the semiarid region of northwestern China.

In this study, field experiments with six fertilization strategies were arranged in three successive years on the Loess Plateau to test the following hypothesis: fertilizing strategies with organic manure increase grain yield and water use efficiency through optimizing soil water and nutrient conditions, and relieved the negative effect of long-term chemical fertilizer on vertical soil water and nutrient cycles based on the analysis of (1) dynamic changes in soil water and nutrients content as well as maize shoot development and yield composition and (2) correlations among grain yield, water use and soil water-nutrients status under additional organic manure application.

## Materials and methods

### Site location

Field experiments were arranged at the Chang Wu Agro-ecological Experimental Station (35°12′30′′ N, 107°40′30′′ E, 1200 m ASL), Chinese Academy of Sciences, located in a typical semiarid area of north-western China. The 30-year average annual temperature is 9.1°C, and the average annual precipitation is 584.6 mm, approximately 70% of which occurs from June to September. The soil is classified as Cumuli -Haplic Kastanozem according to the WRB system (USS Working Group-WRB, 2014), and as Cumuli-Ustic Isohumosols according to the Chinese soil taxonomy system [22, 23]. The contents of soil organic matter, total nitrogen, available phosphorus, and available potassium in the topsoil were 11.8 g kg^-1^, 0.87 g kg^-1^, 14.4 mg kg^-1^ and 144.6 mg kg^-1^, respectively, with a pH of 8.4 and soil bulk density of 1.30 g cm^-3^.

The field study was carried out on the official land which belonged to Chang Wu Agri-ecological Experimental Station, Chinese Academy of Sciences, permission was given after research application passing verification. During the field study none of endangered or protected species were involved. No specific permissions were required for conducting the field study because it was not carried out in protected area.

### Experimental design and treatments

Maize cultivar (Zheng dan 958) was sown in a flat field in April (2012–2014) after soil preparation and harvested in September each year. All plots were 7.5 m long × 4.8 m wide (with 0.8 m buffer zone between plots), with 80 cm of film mulching and 40 cm of un-mulched hedges astride a row of maize plants. Six treatments included three planting densities combined with local- and optimized chemical fertilizer practices (LP, SP and EP), and additional organic manure application (ox manure contained total C, N, K and P of 362.1 g kg^-1^, 20.3 g kg^-1^, 8.5 mg kg^-1^ and 18.2 mg kg^-1^, respectively, with water moisture of 25.4%) at three planting densities as LP-O, SP-O and EP-O treatments, the local practice (LP) was conducted as control variant. The experimental design and fertilizer management are shown in Table 1, and a randomized block design with four replicates was conducted. Water resources relied on natural rainfall rather than irrigation, diseases and insects were controlled using pesticides, and weeds were manually controlled.

**Table 1:** Experimental design and fertilizer management, topdressing *N* as urea (46%, *N*) was applied at the growing stages of jointing (V6), 12^th^ leaves (V12) and kernel blistering (R2), P_2_O_5_ as superphosphate (17%, P_2_O_5_), and K_2_O as potassium sulfate (54%, K_2_O).

Treatments	Experimental design
**LP**	Local practice: basic fertilizer as 135 kg N ha^-1^, 112.5 kg P_2_O_5_ ha^-1^, topdressing 90 kg N ha^-1^ at V6, 60000 plants ha^-1^ with film mulched
**SP**	Super yield practice: 180 kg N ha^-1^, 225 kg P_2_O_5_ ha^-1^ and 225 kg K_2_O ha^-1^ as basic fertilizers, then topdressing 135 kg N ha^-1^ at V6 and 135 kg N ha^-1^ at V12, 90000 plants ha^-1^ with film mulched
**EP**	High yield and efficiency practice: N 150 kg ha^-1^, P_2_O_5_ 150 kg ha^-1^ and K_2_O 150 kg ha^-1^ as basic fertilizers, then topdressing 112.5 kg N ha^-1^ at V6, 75 kg N ha^-1^ at V12 and 37.5 kg N ha^-1^ at R2, 82500 plants ha^-1^ with film mulched
**LP-O**	LP+ organic manure (52.5 t ha^-1^) as basic fertilizer
**SP-O**	SP+ organic manure (52.5 t ha^-1^) as basic fertilizer
**EP-O**	EP+ organic manure (52.5 t ha^-1^) as basic fertilizer

### Sampling and measurements

All fully open leaves of three adjacent plants in each plot were collected at the flowering stage. The length and greatest width of each leaf were measured. Leaf area (*LA*) was then calculated using the formula developed by McKee [24]:

LA=(L×W)×0.75 (1)

Soil water content (*SWC*) was measured gravimetrically both before sowing and after the final harvested. Soil samples were collected between the middle two rows with an auger at 0.1 m intervals to a depth of 0–1.0 m and at 0.2 m intervals to a depth of 1.0–2.0 m, then immediately packed into an aluminum specimen box, and dried at 105°C to a constant weight before calculating the *SWC*.

Topsoil bulk density (*SBD*) was measured using the cutting ring method before sowing in 2012 and after harvest in 2014, with three replicates in each plot. Soil samples were dried at 105°C to a constant weight before the *SBD* was calculated. Meanwhile, all topsoil samples were air-dried and sieved through a 0.15 mm screen to measure soil organic matter content with FeSO_4_ titration methods, total nitrogen content with a Kjeltec-8400 Analyzer Unit (FOSS, the Kingdom of Denmark), and available phosphorus content was measured using the 0.5 M sodium bicarbonate (NaHCO_3_)—molybdenum antimony colorimetric method.

Maize aboveground biomass was measured at the flowering stage. Three adjacent plants were randomly selected and cut at ground level, the shoot samples were inactivated for 0.5 h at 105°C and then oven-dried to a constant weight at 80°C.

At harvest, all cobs of 4.8 m^2^ (four rows, each 2.0 m length) in the middle of each plot were manually harvested to determine final grain yield, expressed at 15% moisture. Cobs were counted and threshed by hand, yield components including the number of cobs in unit area, number of grains per cob, grain number in unit area and 1000-kernel dry weight were measured.

Evapotranspiration (*ET*), water use efficiency (*WUE*) and harvest index (*HI*) were calculated with the following formulas [25]:

ET=ΔSWS+Pi; (2)

SWS=SBD×SD×SWC; (3)

$$WUE=\frac{GY}{ET};$$ (4)

$$HI=\frac{GB}{AB};$$ (5)

where *SWS* is the soil water storage in the 2-m soil depth, *SWC* represents the soil water content (mm), *SBD* represents the soil bulk density, *SD* is the soil depth, *GY* denotes the grain yield, *GB* denotes the grain biomass, *AB* is the aboveground biomass, and Pi is the precipitation during the maize growing season.

### Statistical analysis

One-way ANOVAs were used to evaluate the effects of manure application on maize leaf growth, biomass accumulation, grain yield and *WUE* in SPSS Statistics 17 (IBM, USA) at the 0.05 / 0.01 significance level. Means exhibiting significant differences between treatments, between years and between treatments within each year were separated using Duncan’s multiple comparison analysis at the 0.05 level.

## Results

**Fig 1:**
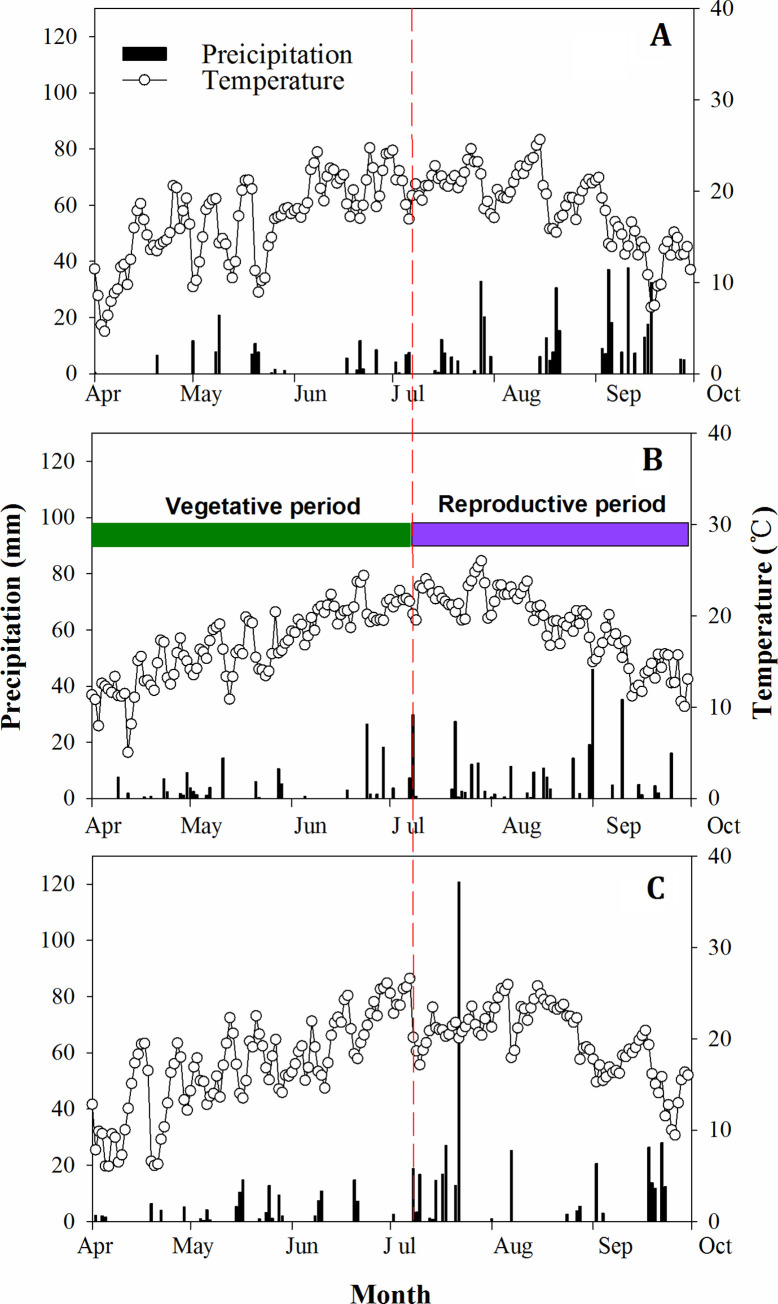
Daily precipitation and temperature over three growing seasons: 2012 (A), 2013 (B) and 2014 (C).

During the three experimental years, the annual rainfall from 2012 to 2014 was 429.8, 440.0, and 503.1 mm, respectively (Fig 1). Approximately 50–70% of the precipitation during the growing season occurred from July to September, which indicated that the water requirement of dryland maize well synchronized with the rainy season.

### Variation in soil bulk density (SBD), total N (N), available P (P) and soil organic matter (SOM) at harvest

**Fig 2:**
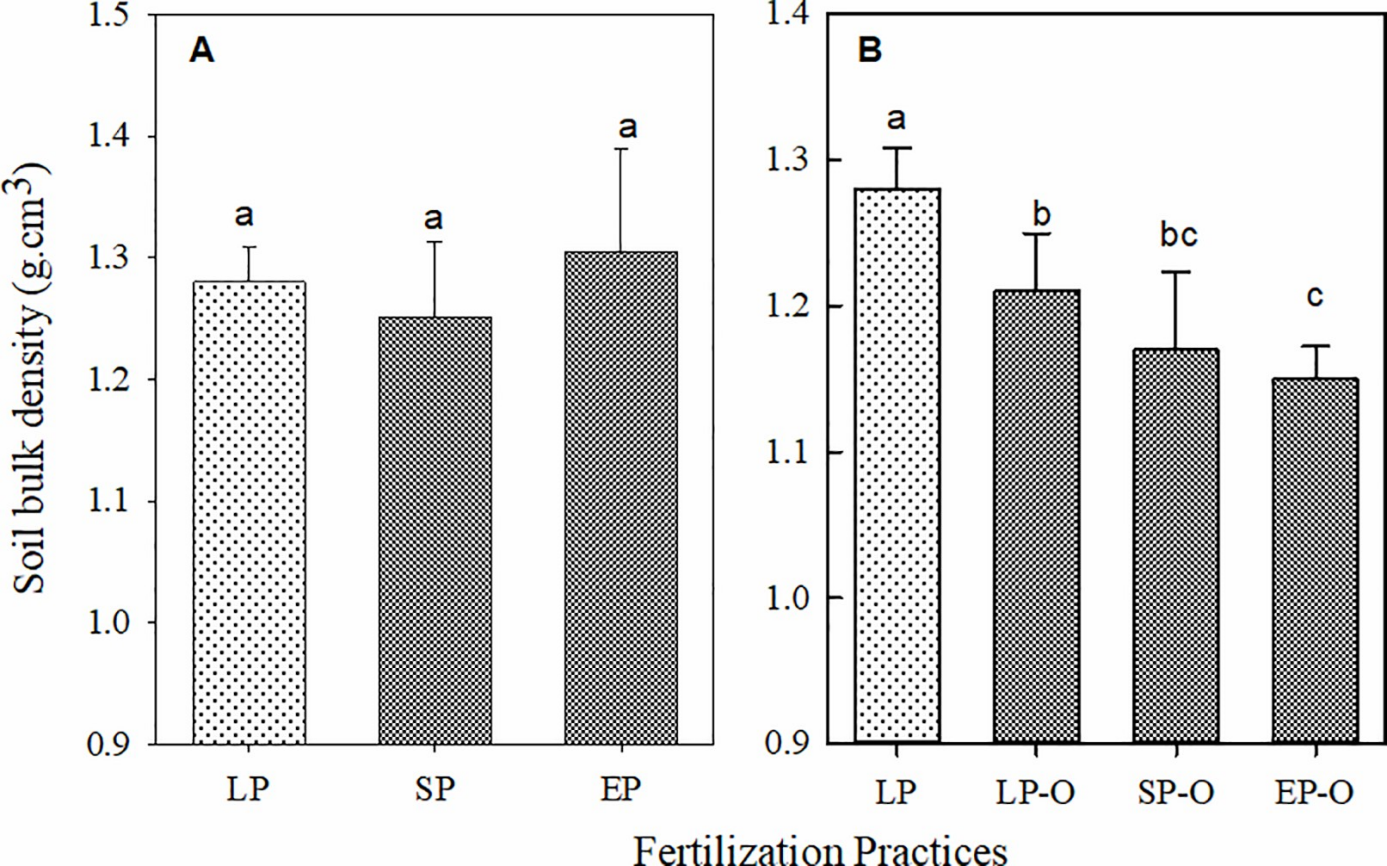
Significant variation in soil bulk density (*SBD*) under chemical fertilization (A) and additional organic manure input (B). *SBD* of six fertilization practices also measured at harvest in 2014. The different letters (a, b and c) above the histograms indicate significant differences at *P* < 0.05, n = 4.

Optimized chemical fertilizer management (SP and EP) exhibited non-significant effects on *SBD* (Fig 2A). The combination of chemical topdressing with basic organic manure benefitted the topsoil structure after three consecutive years, which was supported by the significant *SBD* decrease in LP-O, SP-O and EP-O compared to that in LP (Fig 2B).

**Fig 3:**
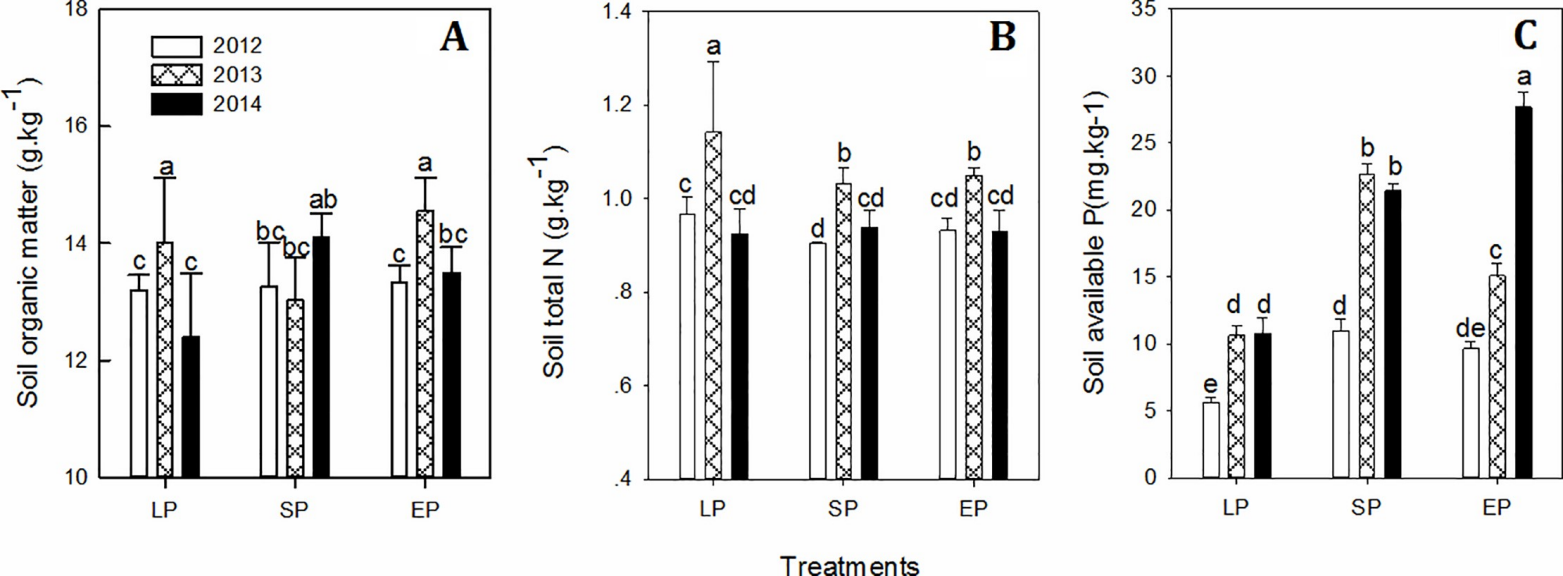
Changes in soil organic matter (A), total nitrogen (B) and available P (C) in the topsoil over 2012, 2013 and 2014. Different letters (a, b, c, d and e) above the histograms indicate the significant differences among the three chemical fertilization practices at *P* < 0.05, n = 4.

**Fig 4:**
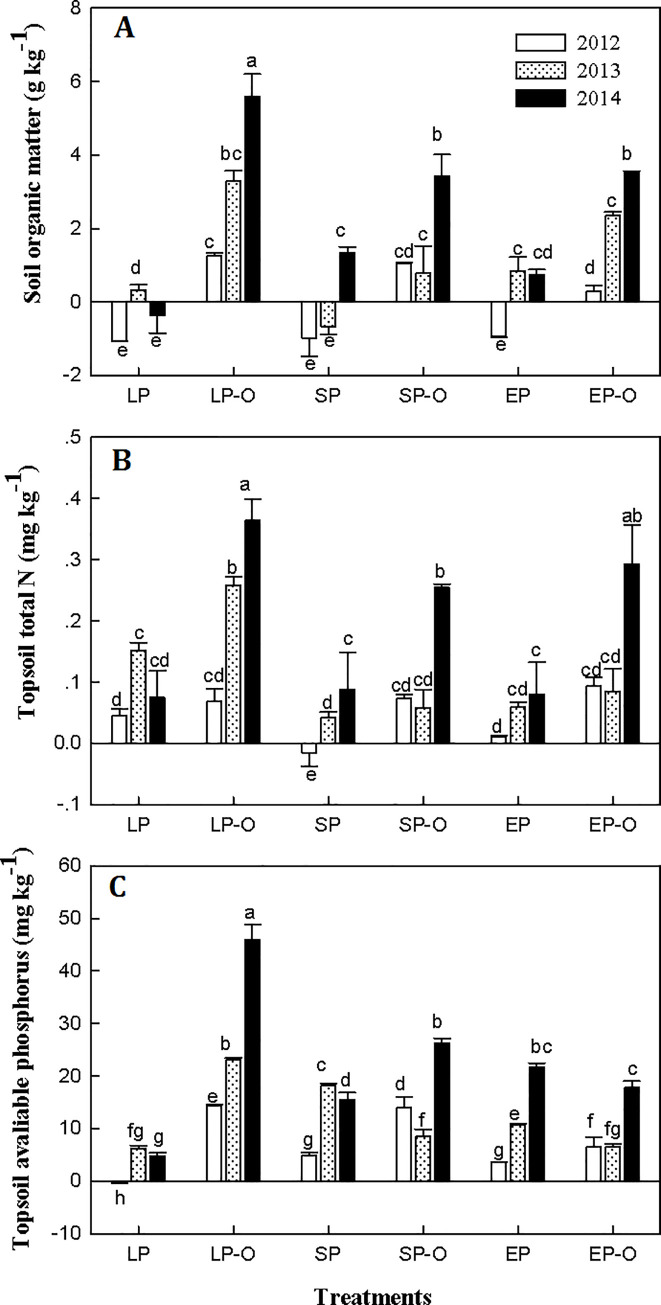
Relative increase of soil organic matter (A), total nitrogen (B) and available P (C) in the topsoil over 2012, 2013 and 2014. Different letters above the histograms indicate the significant differences among the three chemical fertilization practices at *P* < 0.05, n = 4.

SP and EP treatments accorded with maize *N* requirements, but the chemical *P* input exceeded the maize demand after consecutive applications (Fig 3), leading to high residual available *P* in the topsoil at harvest. SP and EP in combination with different planting densities showed a non-significant influence on the *SOM* and total *N* contents. However, when basic organic manure was involved, *SOM* significantly increased and benefitted to the maintenance of the total *N* and available *P*. A significant increase in *N* and *P* occurred in LP-O compared to that in LP and showed as increase at each year (Fig 4B and 4C). The highest *N* increase rate with three planting densities appeared in 2014 after organic manure applied over three consecutive years (Fig 4B). Furthermore, increasing planting density could prevent *P* excessive accumulation and decrease topsoil *P* residue.

### Vertical change in soil water content (SWC) in three growing seasons

**Fig 5:**
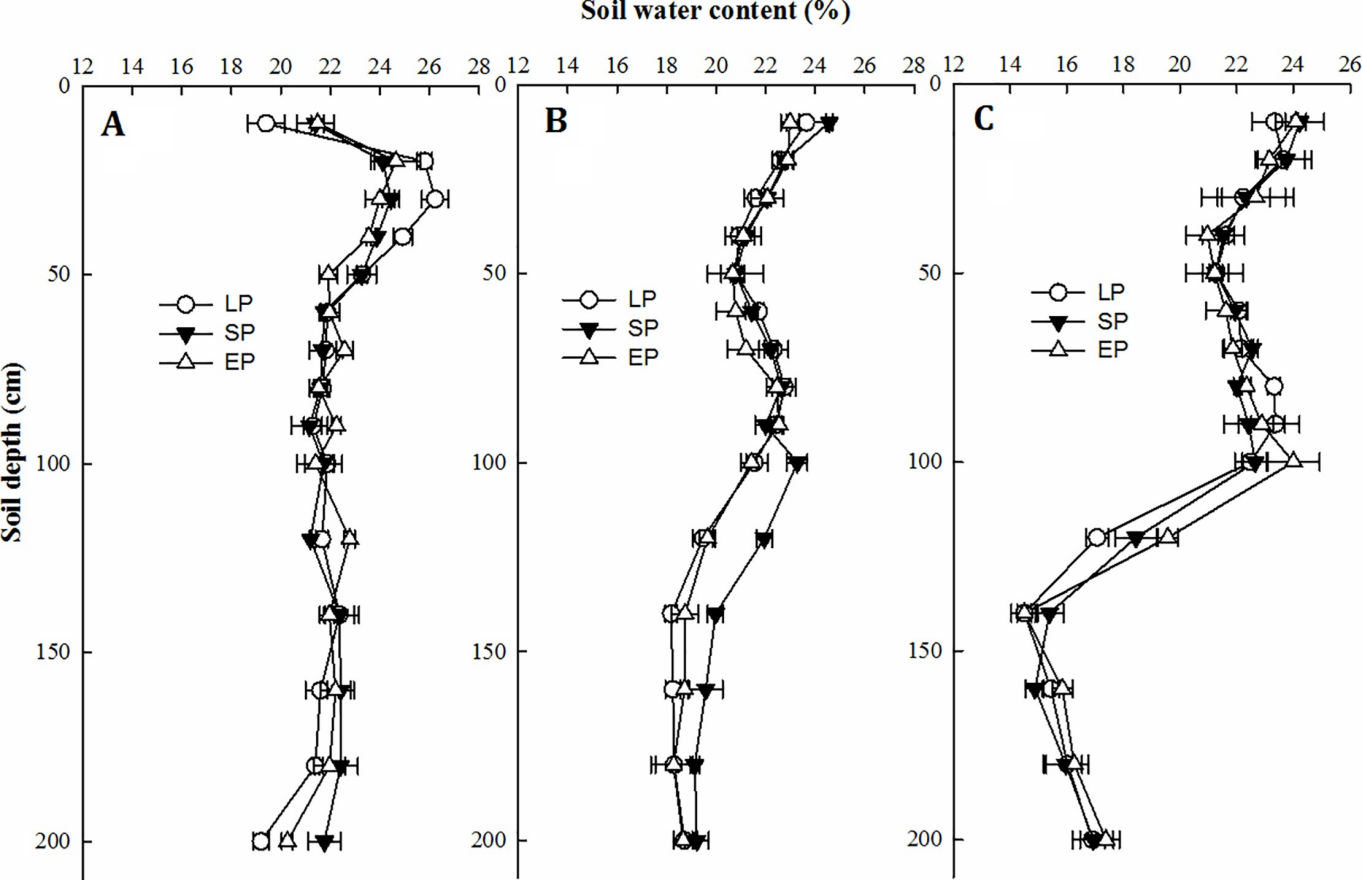
Vertical variation in soil water content at the harvested stage in three growing seasons: 2012 (A), 2013 (B) and 2014 (C).

**Fig 6:**
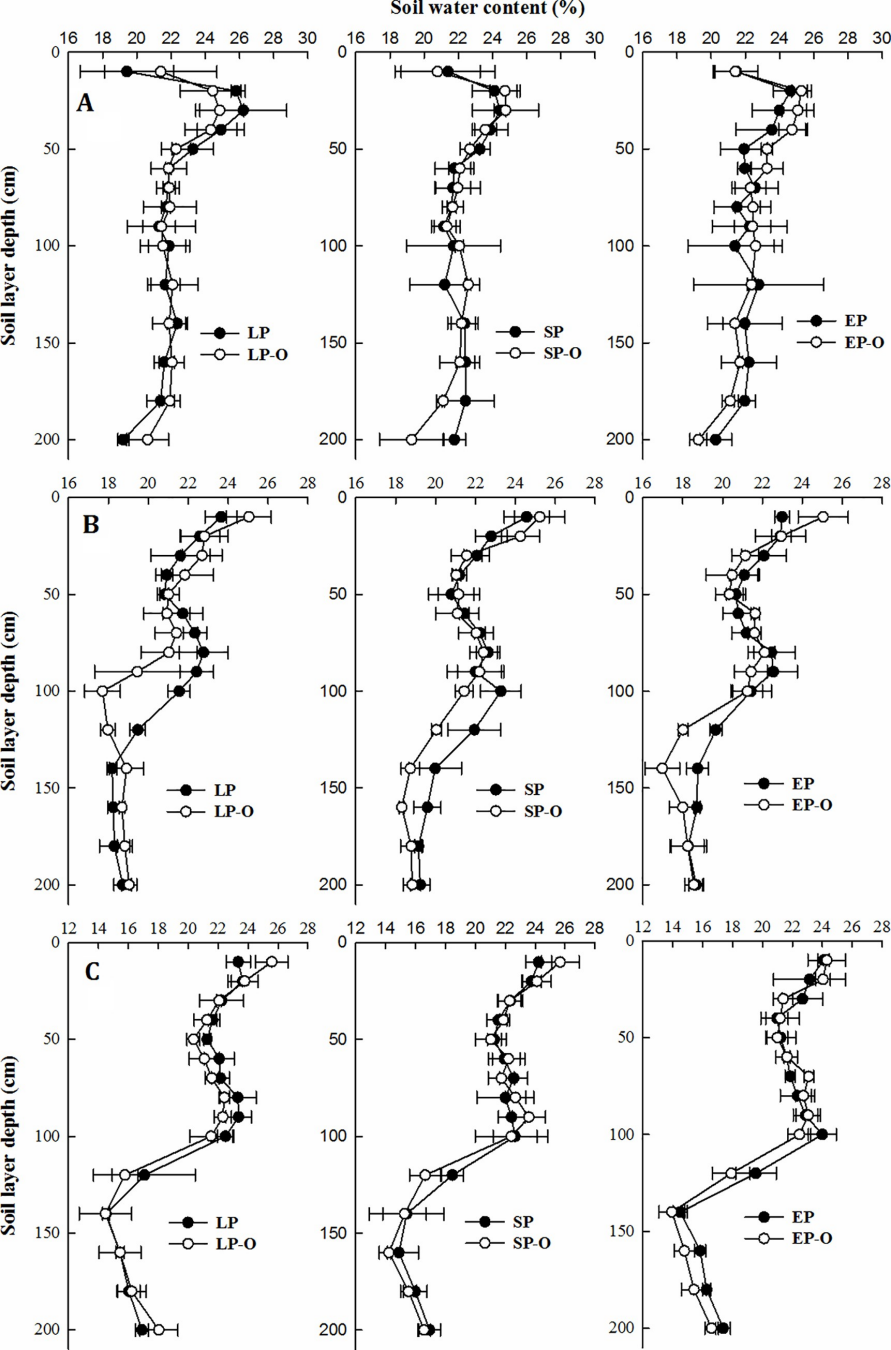
Vertical change in soil water content between the treatments with and without organic manure application in 2012 (A), 2013 (B), and 2014 (C).

*SWC* of the 0–0.5 m and below 2.0 m layers remained stable at approximately 18–24%. However, long-term *N* fertilizer application or continuous application of organic manure significantly decreased the *SWC* in the 1.0–1.5 m soil profile (Figs 5 and 6). Chemical fertilizer combined with organic manure adjusted water infiltration from topsoil to the deep soil layer, and the changed extent was associated with maize planting density (Fig 6). Finally, organic manure maintained *SWC* at a depth of 0–0.5 m at approximately 24%, and *SWC* below 2.0 m was maintained at approximately 18%. Additionally, the soil water use at a depth of 1.0–1.5 m was improved and positively responded to the amount of precipitation.

### Leaf area (LA), aboveground biomass and yield components

Mean *LA* of individual maize plant increased in response to organic manure and decreased with planting density increase. The *LA* of LP-O, SP-O and EP-O increased by 5–18% compared to that under the treatments without manure application (Table 2). The aboveground biomass at maize flowering stage significantly increased by 14 and 36% in LP-O and SP-O, compared to that in LP and SP. At three planting densities, organic manure increased aboveground biomass with different extents by 28%, 29% and 16%, over three years, respectively.

**Table 2:** Leaf area, aboveground biomass at the maize flowering stage, yield components, harvest index (HI) and evapotranspiration (ET) of maize over three growing seasons under chemical fertilization combined with organic manure.

Treatments		Leaf area m^2^ plant^-1^	Aboveground biomass g plant^-1^	Number of cobs m^-2^	Number of grains cob^-1^	Grain number m^-2^	Kernel dry weight (g)	HI (g g^-1^)
				CN	GC	GA	KDW	
Basic chemical fertilizer	**LP**	0.7 ab	108.1 bc	5.8 b	617.1 a	3541.1 b	179.4 ab	0.42 cd
	**SP**	0.6 b	107.1 c	6.8 ab	593.0 ab	4013.3 ab	168.7 bc	0.44 bc
	**EP**	0.6 b	106.1 c	7.7 a	578.1 b	4419.6 a	156.4 c	0.47 b
Basic chemical and organic fertilizer	**LP-O**	0.8 a	138.4 a	5.9 b	612.8 ab	3568.3 b	185.0 a	0.38 d
	**SP-O**	0.7 ab	138.5 a	7.2 ab	612.8 ab	4390.5 ab	180.6 a	0.47 b
	**EP-O**	0.7 ab	123.5 ab	8.2 a	585.7 b	4709.9 a	162.2 c	0.53 a
Source of variation	
Treatments (T)		NS	*	*	*	*	*	*
Year (Y)		*	**	**	*	*	*	*
Y × T		*	*	*	*	*	*	*

* *P* < 0.05

** *P* < 0.01; NS means no significant difference; the value followed by different letters in a column means the significant differences at *P* < 0.05, n = 4.

Additional organic manure input increased the maize cob number per unit area (*CN*), grain number per cob (*GC*), grain number per unit area (*GA*) and kernel dry weight (*KDW*) compared to that of chemical fertilizer applied alone (Table 2). Organic manure applied with different chemical fertilizer strategies increased *CN* and *GC* significantly in SP-O and EP-O compared to that in LP. However, over all treatments, both *GA* and *KDW* decreased with planting density increase. Under integrated effect of organic manure and planting density, *CN* and *GC* increased by 5% and 8%, respectively, compared to chemical fertilizer application alone, and the largest increase in *GA* occurred in EY-O, while *KDW* increased by the largest rate of 7% over three experimental years.

Mean *HI* increased in the three *N* fertilizer topdressing practices (LP, SP and EP), and additional organic manure application enabled a significant and sustainable increase in *HI* compared to only chemical fertilizer application (Table 2). SP-O and EP-O had higher *HI* values than LP-O and other treatments without organic manure addition. The significant increase in *HI* in EP-O resulted from the integrated positive effect of *N* fertilizer topdressing and additional organic manure input.

### Grain yield and water use efficiency (WUE)

Compared to LP (grain yield, 10.7 Mg ha^-1^), maize yields in SP and EP increased by 13.7% and 23.2% over three years (Table 3), which was positively associated with planting density. Meanwhile, with additional organic manure applied, maize yields increased by 22.5% and 31.1%, respectively. From low- to high planting densities, the mean yield increased by 12.2%, 7.9% and 6.7% with additional organic manure, compared to that with chemical fertilizer input only. The contribution of organic manure to yield was 100.0%, 35.3% and 21.4% at the three planting densities. Consequently, combination of *NPK* basic fertilizer with organic manure ensured a sustainable increase in dryland maize yield.

**Table 3:** Grain yield of maize under chemical fertilization strategies combined with organic manure over three experimental years.

Treatments		Grain yield (Mg ha^-1^)			
		2012 (%)	2013	2014	Mean
Basic chemical fertilizer	**LP**	8.4 c	11.4 c	12.4 c	10.7 b
	**SP**	9.6 b c (13.4)	11.9 c (4.2)	15.3 ab (23.5)	12.2 ab (13.7)
	**EP**	11.7 ab (38.7)	12.6 b c (10.9)	14.8 b (20.0)	13.1 a (23.2)
Basic chemical and organic fertilizer	**LP-O**	8.9 b c (5.3)	12.1 ab (6.4)	15.4 a (24.7)	12.2 ab (12.2)
	**SP-O**	10.6 ab (26.1)	13.0 ab (14.3)	15.7 a (27.2)	13.1 a (22.5)
	**EP-O**	12.1 a (43.1)	13.7 a (19.9)	16.1 a (30.4)	14.0 a (31.1)
Source of variation					
Treatments (T)		*			
Year (Y)		**			
Y × T		*			

The values followed by different letters in a column indicate significant differences at *P* < 0.05/0.01, n = 4

* *P* < 0.05

** *P* < 0.01. The percentage (%) in brackets indicates the extent of increase compared to TP, the same as Table 4.

Mean *WUE* in SP and EP significantly increased by 18.6% and 22.5%, compared to that in LP (Table 4). The increase extent in *WUE* resulting from additional organic manure input was 9.1% (LP-O), 4.5% (SP-O) and 3.9% (EP-O) at three planting densities over three consecutive years, which indicated the significant contribution of organic manure to *WUE* as 100.0%, 19.3 and 14.4% at the three planting densities.

**Table 4:** Water use efficiency (WUE) of maize over three growing seasons under combined chemical fertilization and organic manure.

Treatments		WUE (kg ha^-1^ mm^-1^)			
		2012	2013	2014	Mean
Basic chemical fertilizer	**LP**	23.2 c	31.3 b	27.1 c	27.2 c
	**SP**	29.3 b (26.5)	32.7 b (4.6)	33.8 a (24.8)	31.9 b (18.6)
	**EP**	32.4 a (39.9)	35.9 a (14.9)	30.6 b (12.8)	33.0 a (22.5)
Basic chemical and organic fertilizer	**LP-O**	24.6 c (6.0)	32.8 b (5.1)	31.5 b (16.2)	29.6 b (9.1)
	**SP-O**	29.1 b (25.8)	36.7 a (17.5)	34.4 a (27.0)	33.4 a (23.5)
	**EP-O**	33.3 a (43.7)	35.3 a (12.8)	33.8 a (24.9)	34.1 a (27.2)
Source of variation					
Treatments (T)		*			
Year (Y)		**			
Y × T		*			

The values fallowed by the different letters in a column indicate significant differences at *P <* 0.05/0.01, n = 4

* *P* < 0.05

** *P* < 0.01.

## Discussion

Our yields under SP and EP increased by 13.7 and 23.2% compared to local practices, respectively, which was consistent with the successful use of IRNM and ISSM in terms of fertilizer use efficiency and grain yield (increases by 30 and 50%, respectively) in other regions of China [12]. However, the improvement in the soil environment through additional organic manure inputs has generally been ignored in the pursuit of sustainable grain production [26]. The present results indicated that: additional organic manure inputs increased yield to a high and sustainable level, with increases of 12.2%, 7.9% and 6.7% at three densities over three years (Table 3), due to the improvement of soil water-nutrient uptake when additional organic manure applied [27, 28]. Based on the critical water resource situation in the dry-land farming area of China, an improvement in the *WUE* through fertilizer management is an urgent and crucial need [29]. A major factor restricting *WUE* in semiarid regions is the vertical distribution of soil water under limited precipitation, as precipitation is the only water resource that replenishes deeper soil water [17, 30]. In present study, topsoil structure and rain water infiltration into the deeper soil profile of 0.5–1.5 m were improved under organic manure input, as evidenced by the stable *SWC* in the 0–0.5 m and below the 1.5 m soil layer and the *SWC* decrease in the 1.0–1.5 m soil layer (Figs 5 and 6). Consequently, *WUEs* of SP and EP increased by 18.6 and 22.5% over three planting densities. Furthermore, when extra organic manure applied, *WUE* increased by 9.1%, 4.5% and 3.9% more at the three densities, but the potential of organic manure was inevitably weakened with an increase in maize planting density (Table 4). Zhao et al. [5] confirm that organic manure improves soil porosity and then achieves lower water penetration resistance [31, 32], which could further lead to an increase in soil water infiltration [33].

The combination of soil organic matter loss and compaction when organic fertilization is not used, could be expressed as an increase in *SBD*, and impedes soil water storage and nutrient use [9]. Compared with the 30 years average *SBD* on the Loess Plateau, the consecutive application of chemical fertilizer increased *SBD* significantly in our research (Fig 2). However, when extra organic manure applied, the topsoil structure and chemical-physical properties were improved simultaneously, showing that topsoil bulk density significantly decreased, which provides advantageous soil conditions for the available chemical nutrient cycle and simultaneously improves soil fertility and quality [19, 34]. So that, the negative impacts of high planting density and chemical fertilizer input on soil productivity were mitigated.

Making rational choices in terms of organic manure input as a replacement for chemical fertilizer is important to reduce *N* losses and protect the soil environment while guaranteeing food security under the situation of a rapid decrease in *SOM* and available nutrients [5, 15]. After maize harvest, Extra organic manure applications increased the topsoil organic matter, total *N* and available *P* compared to the use of chemical fertilizer input alone due to the efficient storage of residual chemical nutrients. An increase in *SOM* stimulated the potential of soil water use and nutrient (*N* and *P*) availability (Figs 3 and 4). In agricultural ecosystems, soil *N* is often a limiting factor affecting primary production and *NUE* [7]. Presently, massive *N* inputs not only result in soil pollution but also lower *N* efficiency, causing yield stagnation [4, 5]. Compared with chemical fertilizer input alone, total *N* of topsoil increased significantly when extra organic manure applied over consecutive years. This result is also supported by the improvement in soil *N* storage under the combined application chemical fertilizer with organic manure [19]. In our results, long-term optimized *NPK* not only coincided with the maize requirement in different growing stages but also increased *N* use efficiency while part of nitrogen input was replaced by organic manure (Fig 4B). Zhang et al. [35] report that long-term manure application prevents excess *N* from leaching out and forms a rational sufficient *N* condition in the topsoil. Optimized chemical fertilization strategies with organic manure can maintain the soil nutrient balance with high use efficiency and lower environmental costs [28, 34, 36], providing an option for *N* management in sustainable cropping systems [1].

Until 1980, the low contents of soil *P* was the primary factor limiting crop yield in more than 70% of arable land in China [37]. Our results showed that: SP and EP could resolve this limitation but was accompanied by residual of available *P* in topsoil. Furthermore, additional organic manure input increased the amount of topsoil *P* storage (Fig 4C). Parham et al. [38] indicate that manure *P* is more mobile than inorganic *P* fertilizer. Consequently, available *P* concentration and a positive *P* balance are also obtained from inorganic fertilizer application in conjunction with organic manure, by improving soil microbiological activities and soil ecosystem health [3, 9, 19, 39, 40]. It is advisable to reconsider the amount of *P* supplied in manure when deciding how much *P* fertilizer to apply, better for *P* fertilizer and *P*-containing waste are used as efficient as possible [16].

Leaf area (*LA*), as an important agronomic variable, can better summarize the complexity of maize growth caused by the canopy structure [41], which connects available soil water and nutrients with plant biomass production [28]. Combination of chemical fertilizers with organic manure in our plots showed great potential to increase the *LA* of individual plant compared to that of chemical fertilizer input alone (Table 2). Manure prolongs the green leaves staying and allows for a longer duration of leaf photosynthesis [42, 43], and then leaf evaporation is promoted, which ultimately causes a final increase in ET [44].

Combined chemical fertilization with extra manure and topdressing strategies increased the maize aboveground biomass by 16–29% compared with that under chemical fertilizer application only (Table 2), through high density limitation released and harvest index (*HI*) increase. Among the three growing seasons, the trend of *HI* demonstrated a sustainable positive effect of organic manure on grain development (Table 4), i.e., grain dry matter accumulation was improved [45]. Planting density with rational fertilization strategies adjust biomass allocation into shoots and grains [2]. A genetic point considers that biomass allocation into grains is directly associated with final yield [20, 43]. Our results confirmed that aboveground biomass was increased in all three planting densities with organic manure, and high planting density limitations were only applicable to shoot dry matter and not to grain dry matter (Table 4). Finally indicated that the positive effect of organic manure and planting density caused a high and stable yield level and provided an alternative method for obtaining a high yield.

Previous studies confirm that approximately 50% of the total dry matter obtains during the maize grain filling period and is determined by the soil water and nutrient environment situation [45, 46]. Under the integrated effect of organic manure and planting density in our experiments, increases in *GA* and *KDW* showed great potential to stimulate high and stable grain yields, and the correlation coefficients of yield with *GA* (R = 0.90) and with *KDW* (R = 0.76) were evidenced [47]. *GC* generally increased with decreasing planting density and was accompanied by a decrease in *KDW* [48]. However, the values decreased mostly due to a reduction in growing space and severe resource competition under high planting density. Meanwhile, increases in *CN* and *GA* linked the combination of grain yield with organic manure and high planting densities as a stable productive system (Table 2).

## Conclusions

Optimized chemical fertilizer practices significantly increased maize grain yield and *WUE* but were followed by soil degradation. Additional organic manure inputs not only elevated the yield and *WUE* to a higher and more stable level but also improved the soil water-nutrients situation. First, chemical fertilizer in combination with organic manure decreased *SBD*, which provided a superior soil condition for *SOM*, *N* and *P* storage. High planting density with extra manure application increased the topsoil *SOM* significantly over three consecutive years. *N* and *P* were then efficiently captured in the topsoil and were prevented from leaching out. After chemical fertilizer and organic manure were applied together over three years, the water uptake in the 1.0–1.5 cm soil layer improved. All these changes in soil water and nutrients were responsible for the improvement of maize shoot development and biomass allocation in shoots and grains. Finally, extra organic manure inputs facilitated an improvement in the soil water-nutrient status and guaranteed sustainable and stable increases in grain yield and *WUE*. Nevertheless, much research is required to explore the strategies of manure use and the selection of application rates.
